# Promotion of Keratinocyte Proliferation by Tracheloside through ERK1/2 Stimulation

**DOI:** 10.1155/2018/4580627

**Published:** 2018-07-26

**Authors:** JaeGoo Kim, Yu-Kyong Shin, Ki-Young Kim

**Affiliations:** ^1^Graduate School of Biotechnology, Kyung Hee University, Yongin-si, Gyeonggi-do, Republic of Korea; ^2^College of Life Science, Kyung Hee University, Yongin-si, Gyeonggi-do, Republic of Korea

## Abstract

Cell migration and proliferation are important for proper wound healing after skin injury. Recent studies have shown that compounds from plants could promote cell migration and proliferation. Tracheloside, which is a plant lignan, has been found to promote the growth of HaCaT cells over 40% compared to other compounds tested based on a cell proliferation assay. An* in vitro* scratch assay confirmed the healing activity of tracheloside (more than 2-fold increased healing activity after 24 hours of treatment compared with the control) and revealed that this activity is better than that of allantoin (1.2-fold increased after 24 hours of treatment compared with the control), a positive control. With western blot results, wound healing with tracheloside occurred through the phosphorylation of ERK1/2. Therefore, tracheloside is a good candidate to promote wound healing and could be developed as a therapeutic agent for wound treatment or used as a leading compound with higher activity.

## 1. Introduction

Skin wound healing is a complex process involving the reepithelialization of missing cellular structures and tissue layers through three phases: inflammation, proliferation, and remodeling. Of the many cell types required during the wound healing process, keratinocytes are important for epithelialization in the proliferative phase as they are the predominant cell of the outermost layer of the skin. In addition, complex interactions and cross communication between keratinocytes and other cell types during all three phases of wound healing are critical for successful wound closure and repair [1, 2].

As keratinocytes proliferate and migrate toward the upper layers of the epidermis, they are differentiated and transformed through different cell layers to reach their final maturation stage [3]. Cell proliferation is activated by growth factors and cytokines that are released into the injury site. Combined key events such as signaling, cytoskeletal reorganization, and adhesion processes are required [4].

Mitogen-activated protein (MAP) kinase family members such as ERK1/2, JNK, and p38 are well known for their importance in wound healing, cell survival, differentiation, and proliferation [5, 6]. The major mechanism in these processes is the regulation of cell cycle entry and progression. For example, ERK1/2 regulates cyclin D1, which controls cell proliferation [7, 8].

Tracheloside, which belongs to plant lignans, is a component of* Trachelospermum jasminoides* used as herbal medicines in Japan, China, and Korea. It is already known that plant lignans have various effects related to growth factor actions, steroid biosynthesis, cell differentiation, cell transformation, and proliferation [9–11].

In the present experiment, we showed the effect of tracheloside on keratinocyte proliferation. To confirm this effect, we used a scratch wound healing assay, cell proliferation assay, and western blot analysis for signaling related to cell proliferation.

## 2. Materials and Methods

### 2.1. Chemicals

**Figure 1:**
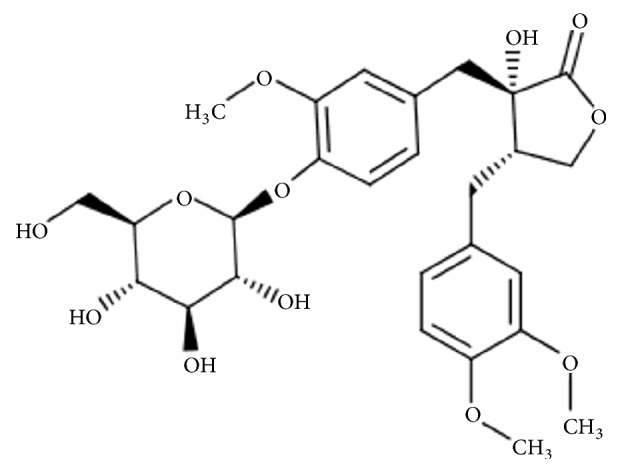
Structure of tracheloside.

All plant extracts were purchased from ChemFaces (Wuhan, China), and the tracheloside chemical structure is shown in Figure 1. Allantoin as a positive control was obtained from Sigma-Aldrich (St. Louis, MO, USA) [12, 13]. The chemicals were dissolved in dimethyl sulfoxide (DMSO), and the stock solutions were stored at -20°C.

### 2.2. Animal Cells and Culture

Human keratinocyte cell line HaCaT were maintained in Dulbecco's Modified Eagle's Medium (DMEM) containing 10% Fetal Bovine Serum (FBS) and 1% penicillin-streptomycin at 37°C in a 5% CO_2_ atmosphere [14].

### 2.3. Cell Proliferation Assay

The proliferation of HaCaT cells by test compounds was tested using a slightly modified cell-based MTT assay [14]. HaCaT cells in DMEM were added to the wells of a 96-well plate at a density of 10^3^ cells per well. Serum-free medium including various concentrations of tracheloside (0, 1, 5, 10, 50, and 100*μ*g/ml) were added and further incubated for 48 hours. MTT (3-(4,5-dimethyl-thiazol-2-yl)-2,5-diphenyltetrazolium bromide, Sigma) in PBS was added into each well at a final concentration of 0.5 mg/ml, followed by incubation for 3 hours at 37°C. The medium was then removed, and cells were suspended in 100 *μ*l DMSO for 10 minutes. Cell proliferation was calculated from optical density (OD_540_) values measured using a microplate reader (BioTek Instruments, Korea) and were reported as a percentage of the vehicle control [14, 15].

### 2.4. *In Vitro* Wound Healing

HaCaT cells were seeded into 6-well plate and cultured to nearly confluent cell monolayers. A linear vertical and horizontal wound was then generated in the monolayer with a sterile 20-200 *μ*l plastic pipette tip. Any cellular debris was removed by washing with phosphate-buffered saline (PBS). Serum-free medium with various concentrations of tracheloside (1, 5, and 10*μ*g/ml) was added in triplicate and incubated for 24 hours at 37°C with 5% CO_2_ atmosphere. Images of the scratched areas were photographed to estimate the relative proliferation of cells at 0 and 24 hours posttreatment. The data were analyzed using an EVOS XL imaging system (Fisher Scientific, USA) by calculating the percentage of scratch closure at each dose point relative to the control. The experiments were repeated three independent times [15].

### 2.5. Western Blot Analysis

Protein was extracted with RIPA buffer and quantified with the Bradford reagent (Sigma). Protein samples with equal amounts (25 *μ*g) were separated by 8-10% SDS-PAGE and transferred onto polyvinylidene fluoride (PVDF, Bio-Rad, USA) membranes. The membranes were blocked with 5% bovine serum albumin (BSA, GenDEPOT, Korea) and then incubated with a 1:2000 dilution of primary antibodies (p38*α*, p-p38, ERK1/2, JNK, p-JNK, and GAPDH from Santa Cruz Biotechnology, CA, USA; p-ERK1/2 from Cell Signaling Technology, MA, USA) overnight at 4°C. The membranes were washed with TBST and incubated with a secondary horseradish-peroxidase-conjugated antibody for 1 hour at room temperature. The membranes were developed using enhanced ECL (Bio-Rad, USA) on a UVITEC imaging system (UVITEC Cambridge, UK). Each experiment was repeated at least twice for consistency of the results [7].

### 2.6. Statistical Analysis

Results are expressed as means ± SD. Statistically significant differences were analyzed with one-way ANOVA with Tukey's post hoc test.

## 3. Results

### 3.1. Enhanced Cell Growth Effect of Tracheloside to Keratinocytes

**Figure 2:**
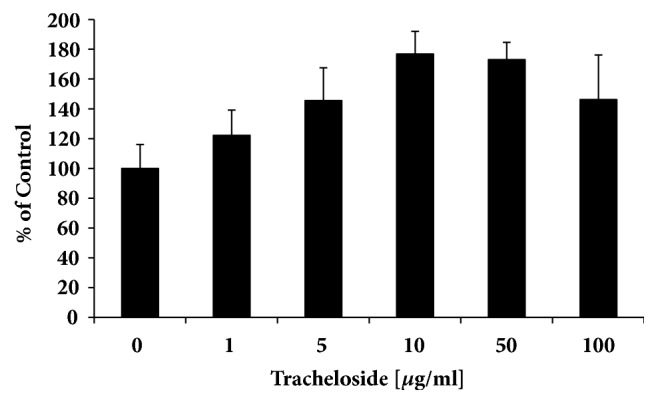
Tracheloside induced the cell proliferation rate. Various concentrations of tracheloside were applied to HaCaT cells cultured for 24 hours in serum-free medium and checked the cell proliferation rate using MTT.

Several available extracts from plants were used to test and compare their effects of proliferation to keratinocyte HaCaT cells (Table 1). It was found that tracheloside increased cell proliferation against human cells (Figure 2).

In 10 *μ*g/ml concentration, HaCaT cells grew over 45.58% more compared to the control.

### 3.2. Tracheloside Increased Wound-Healing

**Figure 3:**
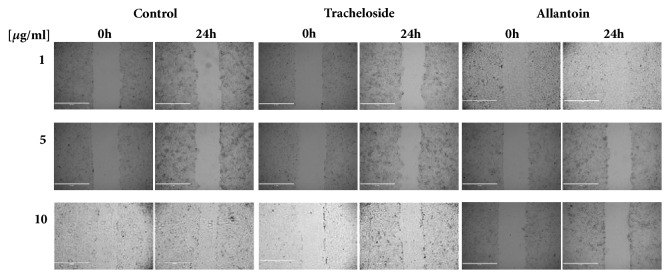
Effect of tracheloside on the proliferation of HaCaT cells through the wound healing assay. HaCaT cells were cultured in a 6-well plate, scratched, and treated with different concentrations of DMSO only, allantoin, or tracheloside. The results were photographed and demonstrated healing of the scratched wound with various concentrations of compounds.

To determine the effect of tracheloside on keratinocyte proliferation, various concentrations of tracheloside were used to treat HaCaT cells. Tracheloside increased the proliferation of HaCaT cells in a dose-dependent manner (Table 2 and Figure 3) compared to the control or allantoin as a positive control. Tracheloside increased the cell proliferation rate by 13.98%, 18.82%, and 17.94% at 1, 5, and 10 *μ*g/ml, respectively after 24-hour treatment. As a result of those findings, 38.14%, 106.13%, and 72.83% increased healing activity was observed, respectively, compared with the control.

### 3.3. Tracheloside Induced ERK1/2 Phosphorylation

**Figure 4:**
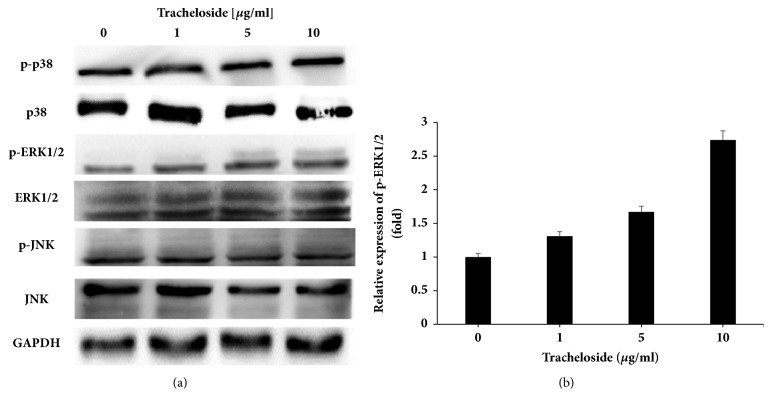
Tracheloside dose-dependently induced ERK1/2 phosphorylation on the HaCaT cells. HaCaT cells were treated with various concentration of tracheloside and the protein was used in western blot analysis. (a) GAPDH was used as a control, and p38, ERK1/2, and JNK, which are related with cell proliferation in their phosphorylated forms, were detected. (b) The p-ERK1/2 was quantified with densitometric analysis and normalized with GAPDH.

Tracheloside treatment promoted the proliferation of HaCaT cells. To investigate whether signaling kinases including p38, JNK, and ERK1/2 participated in proliferation, western blot analysis was performed after treatment with tracheloside (Figures 4(a) and 4(b)). Phosphorylated ERK1/2 increased dose-dependently 1.3-, 1.67-, and 2.73-fold by 1, 50, and 10 *μ*g/ml, but phosphorylated JNK was slightly decreased and phosphorylated p38 did not show any change after treatment with tracheloside.

## 4. Discussion

Tracheloside is a type of plant lignan and an analogue of another plant lignan, arctiin. Arctiin has exhibited some clinical effects including anti-inflammatory, improved immune response to influenza, and antidiabetic activities [16–18]. However, arctiin has also shown antiproliferative effects [19, 20]. Tracheloside is a known antiestrogenic lignan [21]. Other effects of tracheloside have yet to be found.

In the present study, we showed that tracheloside positively affects the proliferation of the keratinocyte, HaCaT, which is comparable with allantoin as the positive control, which exhibited less effect on cell proliferation than tracheloside [12].

ERK1/2, one of the MAP kinase family members, are phosphorylated and activated by MEK, a tyrosine/threonine kinase [22]. Activated ERK1/2 (p-ERK1/2) can change extracellular stimulus to intracellular signal that control gene expression, which contributes to the regulation of cell proliferation [23]. Western blot results show that phosphorylation of ERK1/2 was increased as tracheloside was treated. Therefore, tracheloside affects proliferation through the regulation of ERK1/2 phosphorylation [6, 7].

*In vivo* testing and experiments with epidermal tissue were not performed in this study. These additional data will show a clearer effect of tracheloside in cell proliferation. Based on our research results, tracheloside could be recommended as a lead compound related to wound healing and skin proliferation. Western blot analysis under a pathway of ERK1/2 and RT-PCR results about interleukins will aid in the understanding of how tracheloside stimulates keratinocytes [24–26].

In conclusion, tracheloside can be used as a good candidate to promote wound healing. Furthermore, it could be utilized as therapeutic uses for wound treatment.

## Figures and Tables

**Table 1:** Cell proliferation effect of several plant extracts (% of control).

Compounds	Proliferation rate (100 *μ*g/ml)	Compounds	Proliferation rate (100*μ*g/ml)
Bisdemethoxy-curcumin	2.60 ± 0.70	Falcarindiol	27.71 ± 0.79
Sophoraflavanone G	0.97 ± 0.13	Pimaric acid	81.11 ± 5.35
Acetylshikonin	30.42 ± 5.36	Anwulignan	103.59 ± 1.36
Lobatoside C	2.40 ± 1.25	6,8-Diprenylorobol	3.69 ± 0.05
Alpinumisoflavone	73.56 ± 4.09	Galangin	113.66 ± 3.01
Eupatilin	78.30 ± 1.73	Corosolic acid	72.59 ± 3.27
Kurarinone	10.49 ± 0.49	Tracheloside	145.58 ± 22.1

**Table 2:** Tracheloside increased wound healing after 24 hours of treatment through an i*n vitro* scratch assay.

Concentration of treatment	% of wound healing from 0 hours		
	Control	Tracheloside	Allantoin
1 *μ*g/ml	10.12 ± 1.29	13.98 ± 3.21	13.55 ± 2.38
5 *μ*g/ml	9.13 ± 1.94	18.82 ± 5.95	12.33 ± 2.26
10 *μ*g/ml	10.38 ± 0.19	17.94 ± 2.03	12.50 ± 2.42

## Data Availability

The data used to support the findings of this study are available from the corresponding author upon request.
